# 74-week follow-up of safety of infliximab in patients with refractory rheumatoid arthritis

**DOI:** 10.1186/ar3058

**Published:** 2010-06-22

**Authors:** Isabelle Delabaye, Filip De Keyser

**Affiliations:** 1 Department of Immunology, Schering-Plough nv, 73 Rue de Stalle, Brussels 1180, Belgium; 2 Ghent University, Department of Rheumatology, Ghent University Hospital, De Pintelaan 185, Ghent, B-9000, Belgium; 3 19 Avenue des Vieux Amis, Waterloo 1410, Belgium

## Abstract

**Introduction:**

The objective was to describe the prevalence, types, and predictors of adverse events (AEs) in rheumatoid arthritis (RA) patients treated with infliximab and methotrexate in a daily clinical setting.

**Methods:**

This was a prospective, multi-center, open-label, 74-week observational study in patients with active RA despite treatment with methotrexate and at least one other disease-modifying anti-rheumatic drug. Patients were treated with 3 mg/kg infliximab at weeks 0, 2, and 6 and then every 8 weeks. At weeks 0, 6, 26, 50, and 74, patients answered a health assessment questionnaire, a swollen joint count was made, and adverse events (AEs) occurring during the previous period were registered.

**Results:**

Five hundred and seventy-five patients were treated with infliximab, of which 346 were still on infliximab at the study end, 158 discontinued treatment, and 71 were lost to follow-up. Reasons for discontinuation included safety (n = 74), elective reasons (n = 43), and inefficacy (n = 41). Infusion reactions (n = 33) and infections (n = 20) were the most common AEs causing discontinuation and the most common AEs overall. There were four cases of tuberculosis, all of which occurred in patients negative at screening. Total AEs, serious AEs, and infusion reactions as well as discontinuations for AEs were most frequent during the first 26 weeks. Higher age was a predictor of serious adverse events (SAEs), infection, and discontinuation due to an SAE, but odds ratios were close to one.

**Conclusions:**

AEs and discontinuations due to AEs occur most frequently during the first half year of infliximab treatment in refractory RA patients. The main reasons for discontinuing treatment are infections and infusion reactions. Tuberculosis and other infections remain an important concern in these patients.

## Introduction

Rheumatoid arthritis (RA) is a chronic inflammatory autoimmune disorder of unknown etiology that occurs in approximately 0.8% of the population [1]. Initial therapy for RA has included non-steroidal anti-inflammatory drugs (NSAIDs), ultimately giving way to oral steroids and disease-modifying antirheumatic drugs (DMARDs). More recent practice is to initiate DMARDs early [2-4]. Methotrexate (MTX) has become the DMARD of choice because of its relatively rapid mode of action and good control during prolonged use; however, for many patients, MTX provides only partial relief of signs and symptoms [5].

The development of biological agents targeting the interaction between effector cells has been a major advance in the treatment of RA [1]. Many of these biological agents act by neutralizing TNF-α, which plays a central role in the chronic inflammation and tissue damage of RA [6]. Infliximab is a monoclonal antibody that binds with high affinity and specificity to human TNF and neutralizes its biologic activity [7]. To date, four double-blind, placebo-controlled, randomized studies have been completed in patients with active RA despite DMARD therapy [8-11]. These studies have shown clinical response rates of 40% to 60% in patients treated with a combination of MTX and infliximab.

The most common adverse events (AEs) found in clinical trials of infliximab include upper respiratory tract infection, headache, nausea, sinusitis, rash, pharyngitis, and cough, with infusion reactions (IRs) reported in 5% to 20% of patients [9,12]. Although the clinical trials did not show a significant increase in the risk of infections with the use of infliximab, a meta-analysis of randomized clinical studies found a significantly higher rate of serious infections [13]. Also, some reports have suggested an increased risk of malignancies, especially lymphoma, in RA patients treated with anti-TNF-α therapies [13-15], but this has been refuted by several recent studies [16-18]. Several observational and retrospective studies have shown that, in daily practice, up to one-fourth or one-third of patients discontinue infliximab within one year and that roughly one-third of discontinuations are due to AEs, with IRs the most common type causing discontinuation [19-21].

Here, we performed a multi-center, prospective, observational study on the safety of infliximab in combination with MTX. The aims of this study were to describe the prevalence and types of AEs and identify predictors of AEs and treatment discontinuation. This information should provide expanded data to health care workers and authorities to help estimate and support the appropriate use of infliximab.

## Materials and methods

### Study design and patient selection

This was a prospective, multi-center, open-label, observational study of infliximab in the treatment of patients with active RA despite treatment with MTX and at least one other DMARD. The study was carried out at 77 centers in Belgium between July 2002 and June 2006. The protocol was approved by the ethics committees of the participating study centers, and the study was conducted in accordance with the Declaration of Helsinki. Prior to initiating treatment, written informed consent was obtained from all patients by the treating physician using a form approved by the ethics committees. The study was not registered because it was purely observational and was started in 2002.

Patients eligible for this study had to be diagnosed with erosive RA and have evidence of active disease despite treatment with MTX and at least one other DMARD. Eligible patients also had to be on a stable dose of 15 mg/wk or more of MTX given orally or parenterally for at least three months. Patients with intolerance to MTX despite the association with folic acid could also be included. Additional inclusion criteria were as follows: men or women 17 years or older; 8 or more swollen joints; Health Assessment Questionnaire (HAQ) index of 25 or more (HAQ score × 10 ÷ 6); and absence of tuberculosis demonstrated by simultaneous negative Mantoux test and negative chest X-ray. In the case of a positive Mantoux test or X-ray, the patient had to have had adequate treatment of the tuberculosis for six months before treatment with infliximab. Women of childbearing potential had to be using adequate birth control measures.

Exclusion criteria were as follows: women pregnant, nursing, or planning a pregnancy within two years of enrollment; a history of known allergies to murine proteins; serious infections, such as hepatitis, pneumonia, and pyelonephritis in the previous three months; history of opportunistic infections such as herpes zoster within two months of screening; evidence of active cytomegalovirus, active *Pneumocystis carinii*, drug-resistant atypical mycobacterium, or other opportunistic infections; documented infection with HIV; current signs or symptoms of severe, progressive, or uncontrolled renal, hepatic, hematologic, endocrine, pulmonary, cardiac, neurologic, or cerebral disease; previous or concurrent malignancies, with the exception of surgically cured carcinoma *in situ *of the cervix and surgically cured basal or squamous cell carcinoma of the skin; alcoholism, alcoholic liver disease, or other chronic liver disease; and congestive heart failure grade III and IV.

### Treatments

Treatment was initiated in eligible patients within four weeks of the screening visit. Patients received an infusion of 3 mg/kg infliximab (Remicade^®^; Centocor, Leiden, The Netherlands) at weeks 0, 2, and 6 and then every 8 weeks. Dose escalations or shortening of treatment intervals were not permitted.

### Clinical evaluations

At screening, demographic data and medical history were obtained, and the patient underwent a physical examination, had routine baseline (hematology and chemistry) exams, a chest X-ray, and a Mantoux test. At screening and at weeks 6, 26, 50, and 74 after the start of treatment, patients completed a HAQ [22]. In addition, a swollen joint count (SJC) based on 66 joints was determined [23]. Concurrent medications were recorded at weeks 6, 26, 50, and 74.

### Safety evaluations

Safety data were collected at weeks 6, 26, 50, and 74 for events occurring during weeks 0 to 6, 7 to 26, 27 to 50, and 51 to 74, respectively. A serious adverse event (SAE) was defined as any AE that resulted in death, was life-threatening, resulted in a persistent or significant disability or incapacity, required hospitalization or prolonged a hospitalization, or resulted in a congenital anomaly or birth defect. Also, important medical events that may not have resulted in death, were not life-threatening, or did not require hospitalization may have been considered a SAE when, according to the investigator, they jeopardized the subject or required medical or surgical intervention to prevent one of the outcomes defining a SAE. An IR was defined as any AE that occurred during an infusion, within one hour after an infusion, or was considered by the investigator to be infusion-related. For each time period, the presence or absence of IRs was recorded only once.

### Statistical analysis

Descriptive statistics were used for groups with and without AEs, SAEs, and IRs. T-tests were used to examine differences in the age, age at diagnosis, severity (HAQ index and SJC), the dose of MTX, and the duration of disease between the patients with AEs or SAEs and the patients who had no AEs or SAEs. Fisher's Exact Tests (or chi square tests) were used to look for association between the presence of an AE/SAE and the use of corticosteroids, the use of MTX, and sex. Logistic (stepwise) regression analysis was used to assess the ability of baseline characteristics to predict the manifestation of AEs, SAEs, or infections. Fisher's exact test was used to examine the association between the use of corticosteroids and the manifestation of an infection. Means are presented ± standard deviations, and odds ratios are given with 95% confidence intervals (CI).

## Results

### Baseline demographics and patient disposition

**Figure 1:**
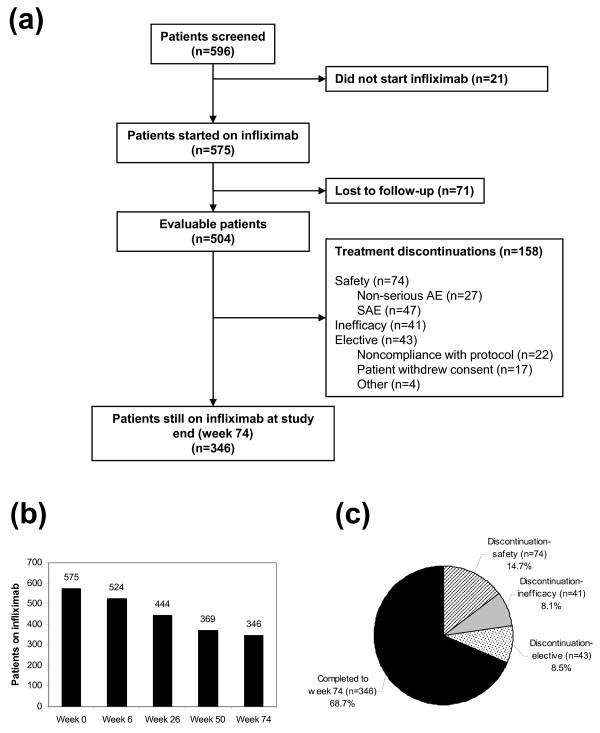
**Patient disposition**. **(a) **Flow chart of patient disposition. A total of 596 patients were screened for this study, of which 575 started infliximab. Of these, 71 were lost to follow-up, so that 504 were evaluated. There were 158 patients that discontinued treatment before week 74, and the remaining 346 completed the study according to protocol and were still on infliximab at study end. 'Other' under treatment discontinuations included three patients that wished to become pregnant and one that withdrew prior to an elective surgery. **(b) **Patients remaining on infliximab at each visit. **(c) **Fraction of evaluable patients completing the study or discontinuing for safety reasons, inefficacy, or elective reasons.

A total of 596 patients were screened for this study. Of the 596 screened patients, 575 started infliximab. There were 71 losses to follow-up, so that 504 patients were evaluable (Figure 1a). Of these, 158 discontinued infliximab before week 74, and the remaining 346 patients were still on infliximab according to protocol at week 74.

The baseline demographics of the patients receiving at least one dose of infliximab are shown in Table 1. Nearly three-quarters of the patients (n = 419; 72.9%) were female. The mean age of patients was 57 ± 13 years, and the mean duration of disease was 10.3 ± 9.4 years. The mean HAQ index at screening was 56.1 ± 15.4, and the mean SJC at screening was 16.3 ± 7.5. Almost all patients (n = 537; 93.4%) were taking MTX, and the mean MTX dose was 14.5 mg/week.

**Table 1:** Baseline demographics of patients receiving at least one dose of infliximab

Characteristic	Value
Age (years) (n = 575)	
Mean ± SD	57 ± 13
Median	58
Range	19 -99
Sex	
Female, N (%)	419 (72.9%)
Male, N (%)	156 (27.1%)
Age at diagnosis (years) (n = 564)	
Mean ± SD	46.2 ± 13.6
Median	47
Range	5-81
Duration of disease (years) (n = 564)	
Mean ± SD	10.3 ± 9.4
Median	8
Range	0-53
HAQ index at screening (n = 573)	
Mean ± SD	56.1 ± 15.4
Median	55
Range	2-98
Swollen joint count at screening (n = 568)	
Mean ± SD	16.3 ± 7.5
Median	14
Range	0-49
MTX doses in mg/week (n = 425)	
Mean ± SD	14.5 ± 3.0
Median	15
Range	5-25
MTX use	
Yes, N (%)	537 (93.4%)
No, N (%)	14 (2.4%)
Unknown, N (%)	24 (4.2%)
NSAID use	
Yes, N (%)	414 (72%)
No, N (%)	46 (8%)
Unknown, N (%)	115 (20%)
Corticosteroid use	
Yes, N (%)	341 (59.3%)
No, N (%)	84 (14.6%)
Unknown, N (%)	150 (26.1%)

HAQ, health assessment questionnaire; MTX, methotrexate; NSAIDs, nonsteroidal anti-inflammatory drugs; SD, standard deviation.

### Efficacy of therapy

Efficacy of the treatment was assessed using the HAQ and by counting the number of swollen joints. The mean HAQ index decreased from 56.0 ± 15.4 at baseline to 29.0 ± 21.4 at week 74 (P < 0.0001), and the SJC decreased from 16.3 ± 7.5 at baseline to 3.2 ± 4.0 at week 74 (P < 0.0001).

### Adverse events

**Figure 2:**
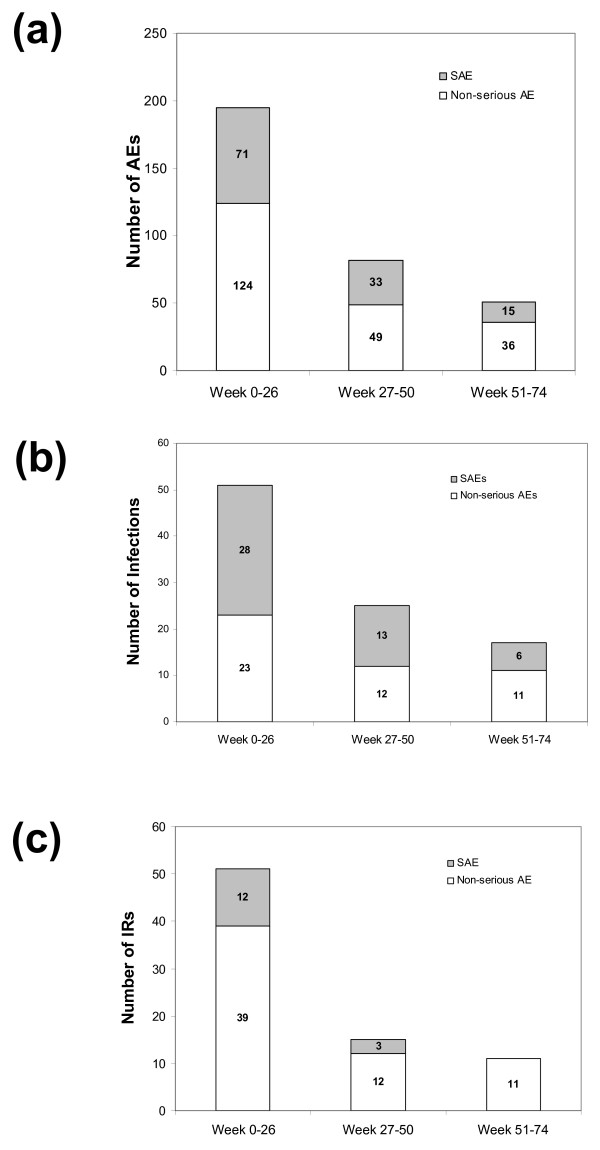
**Incidence of (a) AEs, (b) infections, and (c) IRs during the study**. The incidence of **(a) **all adverse events (AEs), **(b) **infections, and **(c) **infusion reactions (IRs) are shown for weeks 0 to 26, 27 to 50, and 51 to 74. The week 0 to 26 values were calculated by summing the number events for weeks 0 to 6 and weeks 7 to 26. For each time period, the presence or absence of IRs was recorded only a single time. However, this did not affect the calculation of the week 0 to 26 value from the week 0 to 6 and week 7 to 26 values.

A total of 338 AEs were registered during the study. Of these, 121 (35.8%) were considered SAEs (Table 2). The highest number of AEs occurred during the first 26 weeks of treatment with infliximab (Figure 2a). Thereafter, the incidence of AEs decreased gradually over time. Similarly, AEs considered serious (SAEs) were most common during the first 26 weeks.

**Table 2:** Types and severity of adverse events

	All AEs	SAEs	AEs Leading to discontinuation
Any	338 (168)	121 (89)	74
Infection	93 (81)	47 (42)	20
Tuberculosis^a^	4 (4)	4 (4)	3
Other bacterial Infection	38 (33)	29 (24)	10
Viral Infection	8 (8)	5 (5)	1
Opportunistic infection^a^	8 (8)	3 (3)	2
Other	35 (32)	6 (6)	4
Infusion reaction^b^	77 (64)	15 (15)	33
Dermatological disorders	40 (33)	2 (2)	4
Cardiovascular disorders	22 (20)	15 (13)	4
RA-related disease manifestations	17 (16)	11 (11)	2
Gastrointestinal disorders	17 (16)	1 (1)	-
Respiratory disorders	11 (10)	4 (4)	-
Neurologic disorder	11 (10)	1 (1)	1
Non-RA joint manifestations	8 (8)	4 (4)	-
Tumor^a^	7 (7)	6 (6)	5
Benign	3 (3)	2 (2)	2
Malignant	4 (4)	4 (4)	3
Traumatic event	7 (7)	4 (4)	1
Hematologic disorders	4 (4)	4 (4)	1
Psychiatric disorders	3 (3)	1 (1)	2
Liver toxicities	2 (2)	1 (1)	-
Other	19 (16)	5 (5)	1

Values are numbers of AEs, with the number of patients affected shown in parentheses.

^a ^See Supporting information in Additional file 1 for further details.

^b ^Individual infusion reactions are described in Table 3.

AE, adverse event; RA, rheumatoid arthritis; SAE, serious adverse event.

As detailed in Table 2, infection was the most commonly reported type of AE (n = 93), followed by IRs (n = 77). Other common (>5%) AEs included dermatological reactions (n = 40), cardiovascular disorders (n = 22), RA-related disease manifestations (n = 17), and gastrointestinal disorders (n = 17). The most common SAE was infection (n = 47), followed by IRs (n = 15), cardiovascular events (n = 15), and RA-related disease manifestations (n = 11).

There were 93 reported infections in 81 patients. Infections were most common during the first 26 weeks of the study (Figure 2b). Approximately half (n = 47) of the infections were reported as SAEs (Table 2). For cases where the infectious agent was known, the most common type was non-tuberculosis-type bacteria (n = 38). Tuberculosis accounted for four of the infections (three confirmed, one suspected), all of which were considered SAEs. One was a confirmed case of unspecified tuberculosis in a 28-year-old woman who had been exposed to a family member with an overt tuberculosis infection. The remaining two confirmed cases were pulmonary tuberculosis in a 38-year-old woman and a 43-year-old man who did not have known exposure to overt tuberculosis. The fourth case was suspected tuberculosis meningitis in a 69-year-old man that was not confirmed by laboratory tests and later had a differential diagnosis of viral meningitis. All four reported cases of tuberculosis were in patients taking corticosteroids at baseline. Further details about the cases of tuberculosis are presented in the Supporting information in Additional file 1.

The 77 reported IRs occurred in 64 patients. Fifteen of these IRs were recorded as SAEs, and 33 led to treatment discontinuation. Overall, the highest incidence of IRs and the highest incidence of IRs leading to discontinuation occurred during the first 26 weeks (Figure 2c). The most common specific symptoms associated with IRs were allergic skin reactions (n = 24) and hemodynamic events (n = 16; Table 3). Both allergic skin reactions and hemodynamic events were most frequent during the first 26 weeks (i.e., following infusion at weeks 6, 14, and 22). Other common IRs (>5%) included hyperventilation/dyspnea (n = 13), flushing (n = 7), hypertension (n = 6), tachycardia/palpitation (n = 5), and headache (n = 4), all of which were most frequent during the first 26 weeks of treatment.

**Table 3:** Infusion-related events and symptoms

	Week 0 to 6	Week 7 to 26	Week 27 to 50	Week 51 to 74	Total
Infusion-related events^a^	23	28	15	11	77
Symptom					
Allergic skin reaction	2	13	6	3	24
Hemodynamic events (hypotension, syncope, bradycardia, cyanosis)	4	8	2	2	16
Hyperventilation/dyspnea	1	5	5	2	13
Flushing	0	4	2	1	7
Hypertension	2	3	0	1	6
Tachycardia/palpitation	2	2	1	0	5
Headache	1	1	1	1	4
Throat, Quincke's, or mouth edema	0	2	1	0	3
Polyathralgia	2	0	0	1	3
Limb edema	1	1	0	0	2
Flu-like symptom	2	0	0	0	2
Allergic reaction, unspecified	1	0	0	0	1
Other	9	8	5	4	26

^a ^Each infusion reactions could result in multiple symptoms so that the total number of symptoms (n = 112) exceeded the number of infusion-related events (n = 77).

During the study, seven tumors were reported in seven patients (Table 2). Four of the tumors were malignant (one epidermoid epithelioma of the right lung, one lung cancer, one carcinoma *in situ *of the cervix, and one case of chronic myelomonocytic leukemia). Tumors led to discontinuation of treatment in five cases. Further details about these cases are presented in the Supporting information in Additional file 1.

There were 22 cardiovascular disorders reported during the study, 15 of which (68.2%) were considered SAEs. Four of these events led to discontinuation, including one pulmonary thromboembolism, one case of cardiac ischemia, and two fatal events (one myocardial infarct and one cardiac arrest). Of the dermatological and gastrointestinal disorders reported, most (38/40 (95%) and 16/17 (94.1%), respectively) were not considered SAEs.

There were a total of nine deaths during the study, including five for which the main cause of death was infection (three bacterial, one other opportunistic infection, and one unknown type), two due to cardiovascular events (one cardiac arrest and one myocardial infarct), one due to a traumatic event (traffic accident), and one due to a psychiatric disorder (suicide). All patients that died for health reasons were at least 69 years old at the time of death. Details about the deaths occurring during this study are provided in the Supporting information in Additional file 1.

### Treatment discontinuations

**Figure 3:**
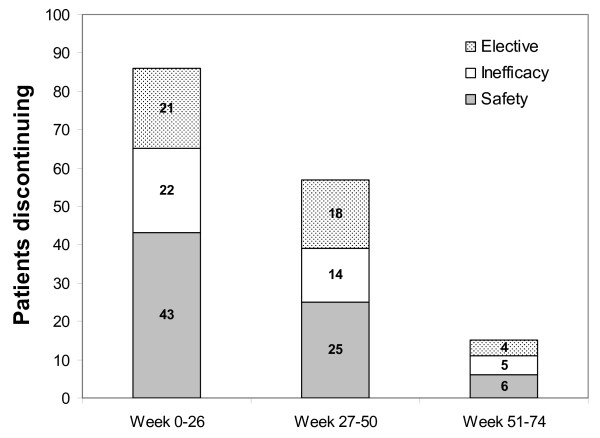
**Treatment discontinuations over time**.

The number of patients remaining in the study at each visit is shown in Figure 1b. Of the 504 evaluable patients, a total of 158 (31.3%) discontinued treatment before week 74, so that the continuation rate at the end of the study was 68.7% (Figure 1c). The leading reason for discontinuation was safety (n = 74; 14.7% of evaluable patients). Discontinuation due to inefficacy occurred in 41 cases (8.1%; Figure 1c). The remaining discontinuations (n = 43; 8.5%) were due to elective reasons, including withdrawal of consent (n = 17), noncompliance with the study protocol (n = 22), wish for pregnancy (n = 3), and wish to stop prior to an elective surgery (n = 1). Of the 74 discontinuations for safety, the majority (n = 47) were for SAEs, although many (n = 27) were for AEs that were not serious (Figure 1a). Treatment discontinuations overall and for safety or inefficacy were most common during the first 26 weeks (Figure 3).

The AEs most commonly leading to discontinuation were IRs (n = 33), infections (n = 20), tumors (n = 5 (2 benign and 3 malignant)), cardiovascular events (n = 4), and dermatological disorders (n = 4; Table 2). There was a significant association between the occurrence of an AE and discontinuation due to the AE for IRs (P < 0.0001), tuberculosis (P = 0.034), tumors (P = 0.0065), and malignant tumors (P = 0.034). Non-serious AEs leading to discontinuation included IRs (n = 21), dermatological disorders (n = 3), neurological (optical neuritis) disorders (n = 1), psychiatric disorders (n = 1), and RA-related symptoms (n = 1).

### Predictors of adverse events, infusion reactions, and treatment discontinuation

Statistical tests were used to determine whether baseline demographics (age at screening, age at diagnosis, duration of disease, HAQ index, SJC, sex, use of corticosteroids, and dose of MTX) were associated with or could predict the manifestation of AEs overall, infections, or IRs or the discontinuation of treatment. Higher age at screening was significantly associated with (P = 0.043) AEs. In addition, higher age was a predictor of the manifestation of AEs (P = 0.0016), infection (P = 0.018), and discontinuation due to an SAE (P = 0.0017), but the odds ratios were all close to 1.0 (1.047 (95% CI, 1.018 to 1.077)), 1.047 (95% CI, 1.008 to 1.087), and 1.076 (95% CI, 1.028 to 1.127), respectively). Neither corticosteroid use nor any of the other baseline variables besides age was associated with or was predictive (P > 0.05) of the occurrence of AEs overall, SAEs, infections, IRs, or discontinuation for AEs, SAEs, IRs, or inefficacy.

## Discussion

This was a 74-week prospective study on the safety of infliximab in patients that had active RA despite treatment with MTX and at least one other DMARD. This study was longer than most randomized clinical trials that have examined the safety of infliximab (mean 0.8 years) [16]. In this study, the most common reason for discontinuing treatment was an AE, of which infections and IRs were the most frequent causes. We also found that AEs as well as discontinuations for AEs most often occurred during the first 26 weeks of treatment.

Our study confirmed that infections are the most common type of AE in RA patients receiving the combination of infliximab and MTX [20,24,25]. Approximately half of all infections were considered SAEs, and infections were also the most common type of SAE. The rate of infections considered SAEs in this study (7.4%; (42 of 575 patients)) was similar to that reported using the same dose of infliximab in ATTEST (Abatacept or infliximab vs placebo, a Trial for Tolerability, Efficacy and Safety in Treating rheumatoid arthritis) (8.5%) [26]. In addition, after IRs, infections were the second leading cause of discontinuation. Also, infections led to five of the nine deaths. The most common type of infection was non-tuberculosis bacteria, although viral infections were also common. This agrees with data from the Swedish practice-based registry ARTIS (AntiRheumatic Therapies In Sweden), which indicate that there is a slight increase in the risk of infection in RA patients treated with anti-TNF-α agents but that it is not driven by any particular type of infection [27]. A recent meta-analysis of randomized clinical trials by Leombruno and colleagues, however, did not find an increased risk of serious infections in RA patients treated with recommended doses of anti-TNF-α therapies [16]. Similar to Takeuchi and colleagues [28], we found that older patients were more likely to have infections, although the odds ratio was close to 1.0. It is also noteworthy that all patients that died from infections were at least 69 years old. Finally, although our study confirms that infections are a reason for concern in refractory RA patients, we cannot determine whether the risk for infection or death due to an infection was increased by treatment with infliximab.

With regard to infections, of particular concern is the increased risk for tuberculosis in patients treated with infliximab, which is generally thought to be due to a lack of compliance with recommendations to prevent reactivation of latent tuberculosis infections [29,30]. In the current study, there were four cases of tuberculosis (three confirmed, one suspected). All four were in patients with negative Mantoux tests and chest X-rays at screening. One of the confirmed cases of tuberculosis appeared to be a new case caused by exposure to a family member with overt tuberculosis. The remaining could have been new cases of tuberculosis, but they may have also been due to latent infections that went undetected by the screening tests [30]. Interestingly, all four cases of tuberculosis were in patients taking corticosteroids at baseline, which could have masked the Mantoux test or caused further suppression of the patient's immune system. Regardless of the reasons for these infections, we concur with the conclusion of Theis and Rhodes [30] that, despite screening and efforts to treat latent infections, clinicians need to carefully monitor for the emergence of tuberculosis infections in patients receiving anti-TNF-α therapies.

In addition to infection, IRs are common in patients treated with anti-TNF-α therapies and are a frequent reason for discontinuation [31]. In this study, IRs were the second-most common type of AE. In nearly half of these cases (42.8%), the IRs caused treatment discontinuation, although, in many cases, the IR causing discontinuation was not considered an SAE. In agreement with Kapetanovic and colleagues [32], age, sex, and HAQ results were not risk factors for IRs. In contrast to their report, however, we did not find an association between IRs and age at diagnosis/onset or longer disease duration.

Some early studies suggest that anti-TNF-α agents may increase the risk of malignancies, especially lymphoma [13-15]. However, this is not supported by a more recent meta-analysis of clinical trial data or more recent data from clinical registries [16-18]. Nevertheless, we paid close attention to the appearance of malignant tumors. There were four cases of malignant tumors, three of which led to treatment discontinuation. However, there were no cases of lymphoma, all four were different tumor types, and there were no obvious relations between the incidence of tumors and any of the patient characteristics.

We also paid close attention to the incidence of cardiovascular AEs because RA patients are at increased risk [33]. Cardiovascular events accounted for 4 of 74 treatment discontinuations, and they were the fourth most common AE overall. They also accounted for two of the nine deaths. Despite the importance of cardiovascular events, there is good evidence that anti-TNF-α therapies reduce the risk in patients with RA to the level in the non-RA population [33-35].

AEs overall, SAEs, and IRs were most common during the first 26 weeks of treatment. We found an association with higher age and the appearance of AEs overall. Also, higher age was a predictor of SAEs, infections, and discontinuation due to a SAE, but the odds ratios were all close to 1.0. Otherwise, we did not identify significant risk factors for AEs overall, SAEs, infections, or IRs in this study.

One of the key aims of this study was to identify reasons for discontinuation in RA patients treated with infliximab. In the evaluable population, the continuation rate at 74 weeks was 68.7%. This is comparable with most other studies of daily clinical practice, which have shown one-year continuation rates between 65% and 73% and two-year continuation rates between 67% and 75% [21,36-38]. The continuation rate in the current study was lower than the one-and two-year rates (91% and 81%, respectively) in a previous multicenter study carried out in Belgium [39]. This difference was partly due to the fact that dose increases were possible in the previous study but not here. In addition, the current study took place after etanercept and adalimumab became available, so that patients had the option of switching to alternative anti-TNF-α therapies. Thus, patients would have been more likely in the current study to discontinue treatment if they or the investigator were uncomfortable with the AEs or the level of efficacy.

Treatment discontinuations were most frequent during the first 26 weeks. The AEs most frequently leading to discontinuation were IRs, followed by infections. Baseline characteristics, including age, did not appear to predispose patients to discontinuation due to an AE. Higher age was a significant predictor of discontinuation due to an SAE, but the odds ratio was close to 1.0. Similarly, Chevillotte-Maillard *et al. *reported no difference in discontinuation rates (median one-year follow-up) or drug survival curves between older and younger patients treated with infliximab [40].

Infections were also most common during the first 26 weeks of the study. This agrees with the findings of the ARTIS study, where the risk of infection was highest in the first year [27]. We suspect that this was due to the discontinuation of susceptible patients rather than an adaptation to the treatment. This is supported by the fact that discontinuation for any AE was most common during the first 26 weeks. Moreover, using data from a registry of British patients, Dixon and colleagues showed that the risk of serious infection is highest in the first six months after the initiation of anti-TNF-α therapies and that the reduction in risk thereafter is associated with physicians excluding patients considered at high risk [41]. Regardless of the reason for the lower risk for infection with time, some risk is always present, so physicians should remain vigilant during the course of treatment with infliximab or any other anti-TNF-α therapy.

Prior to beginning the study, we speculated that the use of corticosteroids would reduce the frequency of IRs and increase the frequency of infections. However, our analysis showed that the use of corticosteroids was not associated with a difference in the likelihood of AEs overall, SAEs, IRs, allergic skin reactions, or infections, nor did it appear to influence the likelihood of discontinuation due to AEs or IRs. These results suggest that patients can continue corticosteroid use during treatment with infliximab, if indicated, without increasing the chance for discontinuation or occurrence of an AE, including infections. The results also suggest that corticosteroids do not prevent infliximab-induced IRs. Notably, all four reported cases of tuberculosis were in patients taking corticosteroids at baseline, and all had negative Mantoux tests at screening. Thus, it is possible that the corticosteroids masked the Mantoux results or increased the risk for tuberculosis infections in these patients by suppressing their immune systems.

## Conclusions

In conclusion, we found that, in RA patients treated with infliximab and MTX, discontinuations and AEs occur most frequently during the first 26 weeks of treatment. The study also emphasizes that physicians should carefully monitor patients for the appearance of infections, including but not limited to tuberculosis and other bacterial infections.

## Abbreviations

AE: adverse event; CI: confidence interval; DMARD: disease-modifying anti-rheumatic drug; HAQ: health assessment questionnaire; IR: infusion reaction; MTX: methotrexate; NSAIDs: nonsteroidal anti-inflammatory drugs; RA: rheumatoid arthritis; SAE: serious adverse event; SJC: swollen joint count; TNF: tumor necrosis factor.

## Competing interests

ID was an employee of Schering-Plough at the time the study was performed and the article was written. FDK received financial support from Schering-Plough and has received research grants from (in alphabetic order): Abbott, Centocor, Roche, Schering-Plough, and UCB.

## Authors' contributions

ID and FDK interpreted and analyzed the data and participated in the writing of the manuscript.

## Acknowledgements

The authors would like to thank Dr. Phillip Leventhal (4Clincs, Paris) for assistance in writing this manuscript, and Mrs. Annelies Vanneuville (Denys Research Consultants bvba, Gent) for assistance in data management, and Mrs. Hermine Leroi for assistance in data analysis.

The members of the REMITRACT study group are Dr. Ackerman C., AZ St-Lucas Gent; Dr. André B., CHU Sart Tilman - Liège; Dr. Badot V., CHU Brugmann - Brussels; Dr. Bailleul Y., CH Institut Bracops site Anderlecht; Dr. Bentin J., RHMS Louis Caty - Baudour; Dr. Berghs H., ZOL - Genk; Dr. Brasseur J.P., Clinique St-Pierre - Ottignies; Dr. Castro S., AZ Maria Middelares St.Jozef - Gent; Dr. Cheroutre G., Polikliniek Bond Moyson - Wetteren; Dr. Coigné E., Jan Yperman Ziekenhuis - Ieper; Dr. Coppens M., ZOL - Genk; Dr. Corluy L., Virga Jesse Ziekenhuis - Hasselt; Dr. Cornet Fr., CHR La Tourelle - Verviers; Dr. Courtois C., Clinique Notre Dame - Tournai; Dr. Coutellier P., Clinique Saint Luc - Bouge; Dr. Dall Armellina S., Clinique Notre Dame De Grace - Gosselies; Dr. Daumerie F., Hôpital de Jolimont - Haine-Saint-Paul; Dr. De Brabanter G., AZ Sint Lucas/Sint Jozef - Assebroek; Dr. De Clercq L., AZ Sint-Augustinus - Wilrijk; Dr. De Decker V., CHU André Vésale - Montigny-le-tilleul; Dr. De Graeve B., Maria Middelares - St.Niklaas; Dr. De Vlam K., Sint Andries Ziekenhuis - Tielt; Dr. Declerck K., Imeldaziekenhuis - Mechelen; Dr. Dhondt E., AZ St-Jan - Brugge; Dr. Di Romana S., CHU St-Pierre - Brussels; Dr. Docquier C., Hôpital de Jolimont - Haine-Saint-Paul; Dr. Dufour JP., UCL Saint-Luc - Brussels; Dr. Dumont M., CH Bois Abbaye et Hesbaye - Waremme; Dr. Durez P., UCL Saint-Luc - Brussels; Dr. Engelbeen J.P., Clinique Ste-Anne/St-Rémi - Brussels; Dr. Fernandez Lopez MJ., CHU Brugmann - Brussels; Dr. Francois D., Clinique Europe St-Michel - Brussels; Dr. Geusens P., ZOL - Genk; Dr. Ghyselen G., OCMW Stadskliniek - Lokeren; Dr. Goemaere S., UZ Gent; Dr. Goethals L., AZ Stuyvenberg - Antwerp; Dr. Golstein M., Hôpital César de Paepe - Brussels; Dr. Gyselbrecht L., Aalsters Stedelijk Ziekenhuis; Dr. Halleux R., Clinique Sainte-Elisabeth - Heusy; Dr. Herman H., AZ Sint Blasius - Dendermonde; Dr. Hermanns P., AZ Maria Middelares - Gent; Dr. Heuse E., Hôpital de la Citadelle - Liège; Dr. Heylen A., Clinique Sainte-Elisabeth - Namur; Dr. Immesoete C., Aalsters Stedelijk Ziekenhuis; Dr. Itzkowitch D., CH Tubize-Nivelle; Dr. Janssens X., AZ St-Lucas Gent; Dr. Jeukens T., CH Bois Abbaye et Hesbaye - Waremme; Dr. Joos R., Jan Palfijnziekenhuis - Merksem; Dr. Kaiser M-J., CHU Sart Tilman - Liège; Dr. Langenaken C., Virga Jesse Ziekenhuis - Hasselt; Dr. Lefebvre S., CH Mouscron Site Refuge; Dr. Lenaerts J., Virga Jesse Ziekenhuis - Hasselt; Dr. Léon M., CHU Ambroise Pare - Mons; Dr. Luyten H., Volkskliniek E.Moyson - Gent; Dr. Maenaut K., Sint Jozefziekenhuis - Malle; Dr. Maertens M., AZ Damiaan - Oostende; Dr. Maeyaert B., AZ Sint Lucas/Sint Jozef - Assebroek; Dr. Martin F., Hôpital de Warquignies - Boussu; Dr. Moens Ph., Cliniques de l'Europe/Ste Elisabeth - Brussels; Dr. Pater C., Clinique St.Joseph - Arlon; Dr. Poriau S., Elisabethziekenhuis - Sijsele; Dr. Praet J., Aalst; Dr. Raeman F., Jan Palfijnziekenhuis - Merksem; Dr. Ravelingien I., Onze Lieve Vrouwziekenhuis - Aalst; Dr. Ribbens C., CHU Sart Tilman - Liège; Dr. Ronsmans I., Clinique Sainte-Elisabeth - Namur; Dr. Schatteman L., AZ Sint-Augustinus - Wilrijk; Dr. Schreiber S., CHU Tivoli - La Louvière; Dr. Stappaerts G., AZ Maria Middelares St.Jozef - Gent; Dr. Stasse P., Clinique St-Joseph - Mons; Dr. Stuer A., Heilig Hart Ziekenhuis - Roeselare; Dr. Van Bruwaene F., Heilig Hart Ziekenhuis - Roeselare; Dr. Van Den Berghe M., AZ Zusters van Barmhartigheid - Ronse; Dr. Van Den Bosch F., Elisabethziekenhuis - Sijsele; Dr. Van den Bossche N., Stadskliniek - St.Niklaas; Dr. Van Essche E., Onze Lieve Vrouwziekenhuis - Mechelen; Dr. Van Wanghe P., Virga Jesse Ziekenhuis - Hasselt; Dr. Vanden Berghe M., Hôpital St- Thérèse - Montignies-Sur-Sambre; Dr. Vanhoof J., ZOL - Genk; Dr. Vanneuville B., Stedelijk Ziekenhuis - Roeselare; Dr. Villers C., CH Grand Hornu; Dr. Volders P., Reuma Centrum - Genk; Dr. Vroninks P., Salvatorziekenhuis -Hasselt;Dr. Walravens M., Mol; Dr. Williame L., AZ Middelheim - Antwerp; Dr. Wouters M., Parc Leopold - Brussels; Dr. Zmierczak HG., Kliniek St-Elisabeth - Zottegem; Prof. Appelboom T., ULB - Hôpital Erasme - Brussels; Prof. Boutsen Y., UCL Mont-Godinne; Prof. De Clerck L., UZ Antwerp; Prof. Devogelaer JP., UCL Saint-Luc - Brussels; Prof. E.M. Veys, UZ Gent; Prof. Houssiau F., UCL Saint-Luc - Brussels; Prof. Mielants H., AZ Sint-Augustinus - Wilrijk; Prof. Peretz A., CHU Brugmann - Brussels; Prof. Steinfeld S., ULB - Hôpital Erasme - Brussels; Prof. Verbruggen G., Prive Praktijk - Izegem; Prof. Verbruggen L., AZ VUB - Brussels; Prof. Westhovens R., UZ Gasthuisberg - Leuven.

## Supplementary Material

Additional file 1 **Supporting information**. This file contains the three supplemental tables. Supplemental table 1 gives details on opportunistic infections during the study, Supplemental table 2 on tuberculosis cases during the study, Supplemental table 3 on tumor cases during the study, and supplemental table 4 on deaths during the study. Click here for file
