# Supplementary material for: 74-week follow-up of safety of infliximab in patients with refractory rheumatoid arthritis
Source: Arthritis Res Ther. 2010 Jun 22;12(3):R121. doi: 10.1186/ar3058 (PMC2911915; doi:10.1186/ar3058)
Supplement: Additional file 1 — Supporting information. This file contains the three supplemental tables. Supplemental table 1 gives details on opportunistic infections during the study, Supplemental table 2 on tuberculosis cases during the study, Supplemental table 3 on tumor cases during the study, and supplemental table 4 on deaths during the study. [file ar3058-S1.DOC]

# Supporting Information

Supplemental Table 1. Types and severity of opportunistic infections

| **Type** | **SAE** | **Non-serious AE** |
| --- | --- | --- |
| Pulmonary aspergillosis | 2 | - |
| Herpes Zoster | 1 | 4 |
| Mycotic skin infection | - | 1 |

Supplemental Table 2. Tuberculosis cases

| **Description** | **Symptoms** | **Concomitant medication** | **Last IFX dose (week)** | **Onset of infection (week)** | **Diagnosis of TB (week)** | **TB confirmed by** | **Mantoux at Screening** | **Chest X-ray at Screening** | **Known exposure to TB**  **since start of IFX** | **Discontinuation due to infection** |
| --- | --- | --- | --- | --- | --- | --- | --- | --- | --- | --- |
| Myoco-bacterium tuberculosis NOS | N/A | Prednisolone 7.5 mg / MTX 15 mg | 15 | 18 | 18 | Positive skin test, sputum culture | Negative | Negative | Yes (daughter) | No |
| Suspected tuberculosis meningitis | Sudden attacks of tremor in the left leg, headache, loss of memory, slow | Methyl prednisolone 4 mg / MTX (dose N/A)/ leflunomide | 28 | 35 | 39 | Not confirmed | Negative | Negative | No | Yes |
| Pulmonary tuberculosis | Fever, loss of weight, dry cough | Medrol 10 mg / MTX 7.5 mg/week | 82 | 85 | 87 | Positive Mantoux (15 mm), sputum culture | Negative | N/A | No | Yes |
| Pulmonary tuberculosis | Constipation, vague abdominal complaints, fever, cough, anorexia, fatigue | Prednisolone 8 mg / MTX 15 mg | 4 | 7 | 11 | Positive Mantoux, chest X-ray, histology | Negative | Negative | Possible from other residents where the patient was living | Yes |

IFX, infliximab; TB, tuberculosis; NOS, not otherwise specified; MTX, methotrexate; N/A, not available

Supplemental Table 3. Tumor cases

| **Description** | **Symptoms** | **Last IFX dose (week)** | **Diagnosis (week)** | **Malignant/Benign** |
| --- | --- | --- | --- | --- |
| Lung epidermoid epithelioma | N/A | 48 | 56 | Malignant |
| Lung cancer | Lumbo ischialgia | 30 | 30 | Malignant |
| Carcinoma in situ of cervix uteri | N/A | 47 | 28 | Malignant |
| Suspicion of breast cancer - confirmed to not be breast cancer but a reactive gland | N/A | 0 | 0 | Benign |
| Benign ganglioneuroma | Latero-cervical mass with pain and pulse on the left | 6 | 10 | Benign |
| Chronic myelomonocytic leukemia | Alteration of general status | 25 | 33 | Malignant |
| Fibromata perdulae | N/A | 74 | Date unknown, reported at week 74 | Benign |

Supplemental Table 4. Deaths

| **Description** | **Concomitant medications** | **Last IFX dose (week)** | **Onset of event (week)** | **Death (week)** | **Comments** |
| --- | --- | --- | --- | --- | --- |
| Pneumonia (bronchitis) | Medrol / Arava | 24 | 49 | 49 | Died due to cardio-respiratory insufficiency |
| Septic shock, pneumonia | MTX 7.5 mg / methyl prednisolone 24 mg | 0 | 0 (3 days) | 0 | 3 days after the first infusion, patient was hospitalized for pneumonia and shock |
| Myocardial  Infarct | MTX 7.5 mg / prednisolone 12 mg | 60 | 68 | 68 | Known cardiac disease with pacemaker |
| Pulmonary aspergillosis | MTX (dose N/A) / methyl prednisolone 10 mg | 23 | 31 | 40 | Patient died due to pneumonia |
| Suicide | MTX 7.5 mg / Prednisolone 7.5 mg | 47 | 48 | 48 | Patient diagnosed with a carcinoma in situ of cervix uteri at week 29 |
| Cardiac arrest | MTX 7.5 mg | 49 | 53 | 53 | Sudden death |
| Road traffic accident (unspecified) | Prednisolone 7.5 mg | 48 | 51 | 51 |  |
| Pneumonia/ septic shock (interstitial lung disease, pancytopenia, acute renal failure) | MTX 15 mg / prednisolone 10mg | 6 | 10 | 14 | Patient received three infusions (weeks 0, 2 and 6) |
| Septic shock from pneumococcal pneumonia | MTX 10 mg/ prednisolone 8 mg | 6 | 7 | 8 | Patient had a history of pneumonia and smoking (stopped 2 years before starting IFX)  Patient received three infusions (weeks 0, 2 and 6) |

MTX, methotrexate; IFX, infliximab; N/A, not available
